# 

**Published:** 1894-11-17

**Authors:** 


					'HWICH HOSPLTAM
No. 42<Moi. xvil WITH EXTRA NURSING SUPPLEMENT.
Satukday, Nov. 17tii, 1894.

				

